# 2015 Middle East Respiratory Syndrome Coronavirus (MERS-CoV) nosocomial outbreak in South Korea: insights from modeling

**DOI:** 10.7717/peerj.1505

**Published:** 2015-12-17

**Authors:** Ying-Hen Hsieh

**Affiliations:** Department of Public Health and Center for Infectious Disease Education and Research,China Medical University, Taichung, Taiwan

**Keywords:** Basic reproduction number, South Korea, MERS-CoV, Nosocomial infection, Outbreak turning point, Mathematical model

## Abstract

**Background**. Since the emergence of Middle East Respiratory Syndrome Coronavirus (MERS-CoV) in 2012, more than 1,300 laboratory confirmed cases of MERS-CoV infections have been reported in Asia, North Africa, and Europe by July 2015. The recent MERS-CoV nosocomial outbreak in South Korea quickly became the second largest such outbreak with 186 total cases and 36 deaths in a little more than one month, second only to Saudi Arabia in country-specific number of reported cases.

**Methods**. We use a simple mathematical model, the Richards model, to trace the temporal course of the South Korea MERS-CoV outbreak. We pinpoint its outbreak turning point and its transmissibility via basic reproduction number *R*_0_ in order to ascertain the occurrence of this nosocomial outbreak and how it was quickly brought under control.

**Results**. The estimated outbreak turning point of *t_i_* = 23.3 days (95% CI [22.6–24.0]), or 23–24 days after the onset date of the index case on May 11, pinpoints June 3–4 as the time of the turning point or the peak incidence for this outbreak by onset date. *R*_0_ is estimated to range between 7.0 and 19.3.

**Discussion and Conclusion**. The turning point of the South Korea MERS-CoV outbreak occurred around May 27–29, when control measures were quickly implemented after laboratory confirmation of the first cluster of nosocomial infections by the index patient. Furthermore, transmissibility of MERS-CoV in the South Korea outbreak was significantly higher than those reported from past MERS-CoV outbreaks in the Middle East, which is attributable to the nosocomial nature of this outbreak. Our estimate of *R*_0_ for the South Korea MERS-CoV nosocomial outbreak further highlights the importance and the risk involved in cluster infections and superspreading events in crowded settings such as hospitals. Similar to the 2003 SARS epidemic, outbreaks of infectious diseases with low community transmissibility like MERS-CoV could still occur initially with large clusters of nosocomial infections, but can be quickly and effectively controlled with timely intervention measures.

## Introduction

From 2012 to June 19 2015, 1,368 laboratory-confirmed human cases of Middle East Respiratory Syndrome Coronavirus (MERS-CoV) in Asia, Europe, the United States, and North Africa have been reported to the World Health Organization (WHO), including at least 487 deaths (World Health Organization , 2015a). Recently, a MERS-CoV outbreak in South Korea emerged when an index case returned home on May 4 after traveling in the Middle East and developed clinical symptoms for MERS-CoV a week later. Subsequent hospitalization in two hospitals respectively between May 15–17 and May 17–20 without sufficient isolation led to numerous nosocomial transmissions in both hospitals.

Although the index case was later tested positive for MERS-CoV on May 20 and subsequently adequately isolated, several secondary clusters of infections in other hospitals caused by patients from this first cluster of infected contacts had already occurred (World Health Organization, 2015b; Cowling et al., 2015), leading to more tertiary transmissions. By July 6, a total of 186 MERS-CoV cases and 36 deaths have been reported, including one case that was confirmed after arriving in China. However, no new case has been reported since, providing indication that the outbreak might be nearing its end. In this work we will make use of a simple mathematical model, the Richards model, to fit the daily epicurve of this outbreak, in order to trace its temporal course, to pinpoint its turning point, and to estimate its transmissibility for nosocomial infections.

## Materials & Methods

### Data

166 South Korean MERS-CoV reported cases by onset date from May 11 to June 14, including one reported in China that we use in this study is obtained from the laboratory confirmed cases in Republic of Korea and China MERS-CoV epicurve, which is publicly accessible from the WHO website (World Health Organization, 2015c) and provided in (Table S1). The onset date for every laboratory confirmed case is available; including the case that was confirmed in China. Cases reported with onset after June 14 were found to have no impact on our quantitative results and hence is not used in this study.

### Richards model

In the context of infectious disease modeling (Hsieh, Lee & Chang, 2004; Hsieh & Cheng, 2006), the analytic solution of the Richards model (Richards, 1959) is of the form

\begin{eqnarray*} C(t)=K[1+{\mathrm{e}}^{-r a(t-{t}_{i}-(\ln a){}r a)}]^{-1{}a}, \end{eqnarray*}

where *C*(*t*) is the cumulative number of laboratory confirmed cases at day *t*, where *t* = 0 is May 11, the starting date of the MERS-CoV outbreak. *K* is the total case number of the outbreak, *r* is the per capita growth rate of the cumulative case number, *a* is the exponent of deviation of the cumulative case curve, and *t_i_* is an outbreak turning point (or the peak), which signifies the exact moment of an upturn or downturn in the rate of increase for the cumulative case number of an outbreak, which is obviously important.

There have been ample studies in literature of epidemic modeling using the Richards model, from earlier applications to plant diseases (e.g., Madden, 1980; Lalancette, Madden & Ellis, 1988; Segarra, Jeger & Van den Bosch, 2001; Hau & Kosman, 2007) to more recently modeling various human diseases (Zhou & Yan, 2003; Caceres, Kumma & Wright, 2010; Mostaço-Guidolin et al., 2012; Wang, Wu & Yang, 2012; Chan, Tuite & Fisman, 2013; Liu, Tang & Xiao, 2015, etc.).

The Richards model is a phenomenological model which describes the growth of the outbreak cumulative case number. Three model parameters of epidemiological importance are *K*, *r*, and the turning point *t_i_* of the epidemic, which can be estimated by fitting the Richards model to the epicurve of the outbreak, using any standard software with nonlinear least-squares (NLS) approximation subroutine, e.g., SAS (which is used in this work) or MATLAB. Applications of the Richards model on various infectious disease outbreaks such as pandemic influenza and HIV can be found in, e.g., Hsieh et al. (2011) or Hsieh (2013).

### Reproduction number

The formula for the basic reproduction number *R*_0_, the average number of secondary infectious cases produced by an infectious case in a totally susceptible population in the absence of specific control measures, is given by *R*_0_ = exp(*rT*), where *r* is the growth rate we estimate from the Richards model fitting and *T* is the serial interval of the disease, or the average interval from onset time of one individual to the onset time of another individual infected by him/her. It has been shown mathematically (Wallinga & Lipsitch, 2007) that, given the growth rate *r*, the expression *R*_0_ = exp(*rT*) provides an upper bound for basic reproduction number over estimates obtained from all assumed distributions of the serial interval *T*. Note also that the Richards model is sometimes given in its differential equation form (Hsieh, Lee & Chang, 2004), which can be fitted to the daily incidence data (i.e., the rate of change of the cumulative data).

## Results

**Figure 1:**
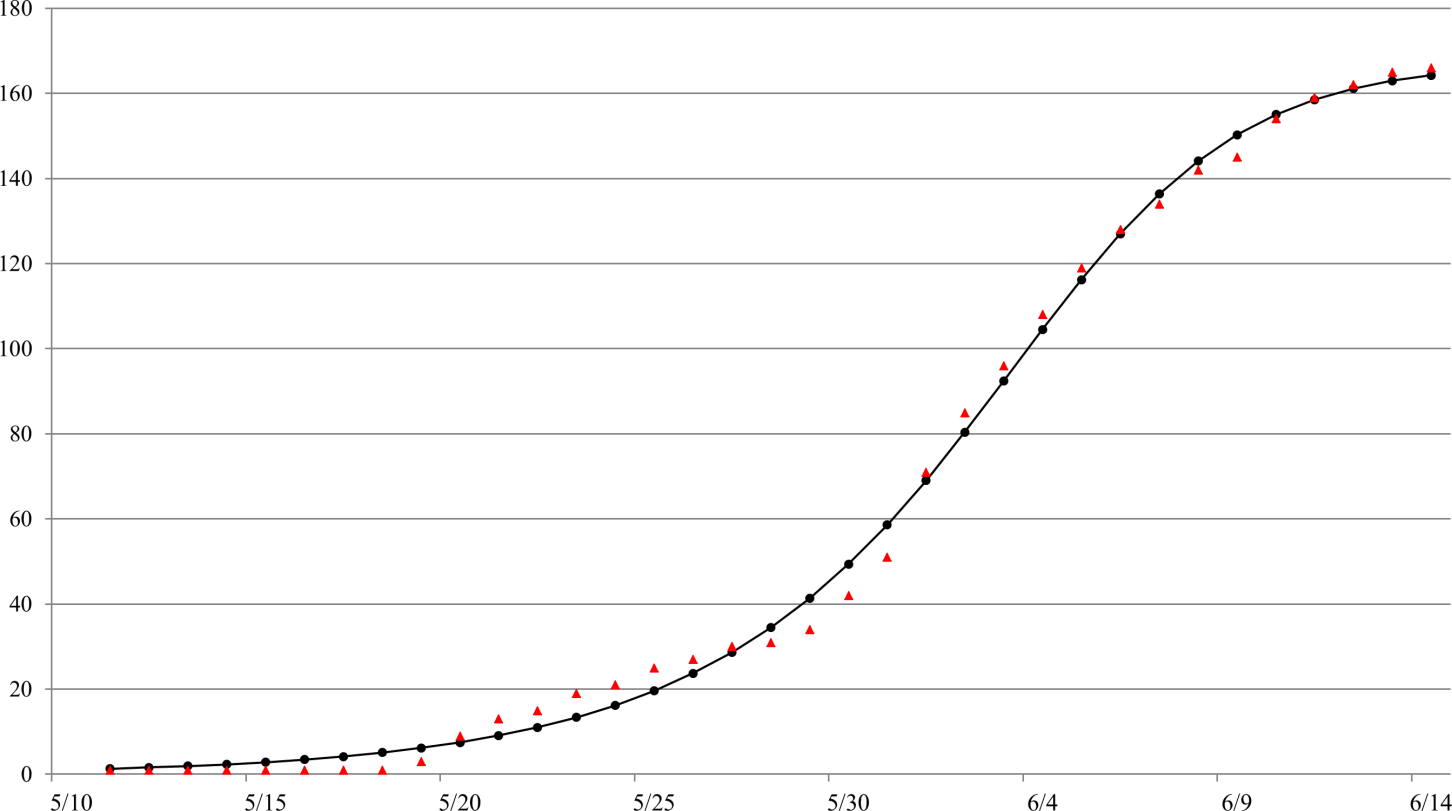
Model fit of the Richards model to cumulative reported MERS case data by onset date in South Korea, May 11–June 16, 2015.ara.

The Richards model provides a good fit to the daily South Korea MERS-CoV case data by onset date from May 11 to June 14 (see Fig. 1), a total of 166 reported cases. The estimated parameters values of the model fit with 95% confidence intervals (CI) are given in Table 1. The parameter estimates and the 95% CIs were obtained using the NLIN subroutine in SAS, a nonlinear least-squared approximation routine. The resulting 95% CI is a measure of uncertainty for the parameter estimation but not for the errors in model fit, which conceivably could be much larger.

The estimated outbreak turning point of *t_i_* = 23.3 days (95% CI [22.6–24.0]), or 23–24 days after the onset date of the index case on May 11, pinpoints to June 3–4 as the time of the turning point or the peak incidence for this outbreak by onset date.

**Table 1:** Summary table of estimated parameter values for model fit of cumulative MERS case data by onset date, 5/11–6/14, 2015, for a total of 166 reported cases, to the Richards model. The 95% CIs of the estimates are given in parenthesis.

Time period	Growth rate *r*	Case number *K*	Turning point *t_i_*	Basic reproduction‘ number *R*_0_
5/11–6/14	0.194 (0.161∼0.226)	167 (160∼175)	23.3 (22.6∼24.0)	11.5 (7.0∼19.3)

For computation of the basic reproduction number *R*_0_, we make use of the point estimate and 95% CI of the serial interval for South Korea MERS-CoV outbreak, *T* = 12.6 days (95% CI [12.1–13.1]), reported by Cowling et al. (2015). Subsequently,we estimate *R*_0_ to be ranging between 7.0 and 19.3.

## Discussion

To locate the turning point of disease transmission, we note that the incubation period of MERS-CoV for this outbreak has been estimated at 6.7 days (95% CI [6.1–7.3]) (Cowling et al., 2015). Therefore, we can deduce that the turning point for *disease infection* had in fact occurred seven to eight days prior to onset; that is, around May 27–29. Interestingly, it was during this exact time period that a yet unobserved second and even larger cluster of at least 70 infections had occurred among patients, visitors, and staff at another hospital due to contact with Case 14 of the first cluster of infections, who was receiving treatment at the emergency room of this hospital (Cowling et al., 2015). Hence, we believe that May 27–29 is the peaking time when the number of infections occurring at the time reaches a maximum and starts to decrease.

We note further that during May 27–29, the 6th–12th cases from the first cluster were tested positive for MERS-CoV, including two health professionals who cared for the index case, while the remaining cases were either patients or their spouse who shared the room with the index patient during May 15–17 (World Health Organization, 2015d; World Health Organization, 2015e). The timing of the laboratory confirmation of these additional cases from the first cluster of nosocomial infections was instrumentally important in providing sufficient evidence of an emerging outbreak. Subsequently, the Korean authority implemented intervention measures for disease control in these hospitals and for contact tracing in the community (World Health Organization, 2015d) with large-scale quarantine and school closings, which led to a decrease in nosocomial transmissions and further prevented its spread to community. Thus, we are able to observe the subsequent downward turning point in case number by onset date during June 3–4.

Basic reproduction number *R*_0_ of an infectious disease differs significantly under different settings and mode of transmissions, which is an important factor to consider when it is used to assess the risk of spread of an infectious disease (Fisman, Leung & Lipsitch, 2014). Our estimate of *R*_0_ for MERS-CoV outbreak in South Korea is significantly higher than earlier estimates of *R*_0_ for MERS-CoV outbreaks in Middle East, (e.g., Breban, Riou & Fontanet, 2013; Cauchemez et al., 2014; Fisman, Leung & Lipsitch, 2014; Poletto et al. 2014; Chowell et al. 2014; Kucharski & Edmunds, 2015; Kucharski & Althaus, 2015; Nishiura et al., 2015), but closer to that of Majumder et al. (2014). It is likely due to the nosocomial nature of outbreak that was studied in Majumder et al. (2014) which is similar to the focus of this study, i.e., the South Korea nosocomial outbreak. It has been suggested that, due to the nosocomial infections that resulted in several large hospital clusters, estimates of basic reproduction number for nosocomial infections and superspreading events (Riley et al., 2003) such as the South Korea MERS-CoV outbreak, where three cases were believed to have infected 24–70 cases, would be misleading and inappropriate (Cowling et al., 2015; Fisman, Leung & Lipsitch, 2014). However, substantial disparity in estimates of *R*_0_ for the same viral disease that we observe for this nosocomial outbreak highlights the importance of settings in quantification of disease transmissibility and the risk involved in cluster infections in crowded settings such as healthcare facilities.

Similar to SARS-CoV virus, MERS-CoV is typically transmitted via direct contact or larger virus-laden droplets that travel only a few meters. Moreover, for nosocomial transmissions such as the South Korea MERS-CoV outbreak, hospital settings tend to promote aerosolization of infectious respiratory droplets or other potentially infectious materials in the hospitals, thereby amplifying transmission (World Health Organization, 2003). During the 2003 SARS epidemic, when nosocomial infections was a major cause of outbreaks in Hong Kong, Singapore and Taiwan (see, e.g., Ho & Su, 2004 or Hsieh et al., 2014), most modeling studies estimated *R*_0_ for SARS in the range of 2–4 (World Health Organization, 2003; Bauch et al., 2005), which is relatively high when compared with other wide-spread respiratory infectious diseases such as influenza. However, with prompt interventions, the 2003 SARS epidemic quickly ended in all affected areas in a matter of a few months and has not emerged again; giving indication that SARS virus also had in fact a very low transmissibility *in community settings* with the exception of the superspreading event at the Amoy Gardens in Hong Kong (Yu et al., 2004). This hypothesis is further affirmed by the recent MERS-CoV outbreak in South Korea, where community spread was effectively prevented with timely intervention measures.

Finally, we note that while using cumulative data tends to have the advantage of smoothing out some of the stochastic variations which often occurs in epidemic data (Hsieh, Fisman & Wu, 2010), cumulative data also has the disadvantage of introducing auto-correlation, potentally leading to biased high estimates of *R*_0_ (Razum et al., 2003). Moreover, model fitting to cumulative disease data could lead to potentially large errors in parameter estimates and subsequently in the corresponding confidence intervals (King et al., 2015), which cannot be overlooked when we attempt to interpret the results of modeling studies. For the purpose of comparison, parameter estimation results of fitting the Richards model to daily incidence data is provided in File S1 which, as can be expected, are very similar to those parameter estimates obtained from the cumulative data (see Table 1), but with larger 95% CI ranges.

## Conclusion

MERS-CoV virus is very similar to SARS-CoV with regard to its potential to spread nosocomially. Therefore timely and effective intervention and control measures in the hospitals, as was implemented in this South Korea outbreak, are essential to quickly contain its spread in hospital settings and to prevent its potential spread to the community, and to other countries in the world.

## Supplemental Information

10.7717/peerj.1505/supp-1 Table S1 South Korea MERS data

10.7717/peerj.1505/supp-2 File S1 The Richards model fitting for daily incidence data

## Additional Information and Declarations

### Data Availability

The following information was supplied regarding data availability: The datset is publically accessible from the WHO website: http://www.who.int/csr/disease/coronavirus_infections/maps-charts/en/.
