# Supplementary material for: 2015 Middle East Respiratory Syndrome Coronavirus (MERS-CoV) nosocomial outbreak in South Korea: insights from modeling
Source: PeerJ. 2015 Dec 17;3:e1505. doi: 10.7717/peerj.1505 (PMC4690341; doi:10.7717/peerj.1505)
Supplement: File S1 [file peerj-03-1505-s002.docx]

**Supplementary Information S2: The Richards model fitting for daily incidence**

The Richards model for daily incidence is given by the differential equation:

Where C′(t) is the time derivative of the cumulative case number C(t).

Fitting the above model to the daily incidence data for South Korea MERS outbreak from 5/11 to 6/14, using the cumulative number of daily incidence up to time t for *C(t)*, yields the following parameter estimates:

**Table S2**. Estimated parameter values for model fit of daily incidence data by onset date, 5/11-6/14, 2015, for a total of 166 reported cases, to the Richards model. The 95% CIs of the estimates are given in parenthesis.

| Time Period | Growth rate  r | Case number K | Basic reproduction number R_0_ | Exponent of deviation a |
| --- | --- | --- | --- | --- |
| 5/11-6/14 | 0.190 (0.121~0.259) | 171  (161~181) | 11.0  (4.3~29.8) | 1.86 (0.49~3.24) |

The figure for the data fitting is given below in Figure S1.

**Figure S1**. Model fit of the Richards model in differential equation form to reported daily MERS incidence data by onset date in South Korea, May 11-June 16, 2015, where the red triangles are the daily incidence data and black dots are the model-predicted numbers:
